# An oral rotavirus-vectored vaccine confers protection against *Clostridium perfringens* and rotavirus

**DOI:** 10.1128/jvi.00178-26

**Published:** 2026-03-26

**Authors:** Jun Wang, Jitao Chang, Zhigang Jiang, Hechun Deng, Qi Jia, Kuanhao Li, Jiangang Zhao, Haoyu Xiang, Fan Yang, Songkang Qin, Yuxin Han, Binxin Yao, Fang Wang, Xin Yin, Dongbo Sun

**Affiliations:** 1 State Key Laboratory of Animal Disease Control and Prevention, Harbin Veterinary Research Institute, Chinese Academy of Agricultural Sciences, Harbin, China; 2 College of Animal Science and Veterinary Medicine, Heilongjiang Bayi Agricultural University, Daqing, People's Republic of China; 3 Institute of Western Agriculture, Chinese Academy of Agricultural Sciences, Changji, China; Loyola University Chicago—Health Sciences Campus, Maywood, Illinois, USA

**Keywords:** rotavirus, *Clostridium perfringens*, enteric viral vector, enteric pathogens, vaccine

## Abstract

The segmented genome of live-attenuated rotavirus (RV) vaccines provides an ideal platform for developing multivalent vaccines against enteric pathogens. Here, we engineered the commercial human RV vaccine strain LLR as a vector to deliver *Clostridium perfringens* alpha-toxin (CPA), a critical virulence factor associated with gastroenteritis. A screen of four 2A peptide sequences identified that porcine teschovirus-1 2A (P2A) preceded by an N-terminal GSG spacer enabled optimal cleavage and antigen expression. In mice, the resulting recombinant virus elicited potent systemic antibody responses against both RV and CPA following oral administration. When tested in a maternal immunization model, intramuscular immunization of cows generated high titers of neutralizing antibodies in colostrum, demonstrating its potential utility for passive immunization. By integrating a key bacterial antigen into a licensed RV vaccine backbone, we establish a versatile strategy to combat viral-bacterial co-infections and polymicrobial enteric diseases, offering a promising new approach to reduce the global burden of diarrheal mortality.

**IMPORTANCE:**

The lack of vaccines targeting polymicrobial enteric infections represents a critical gap in the fight against diarrheal diseases, a leading cause of infant mortality worldwide. To bridge this gap, we engineered a novel bivalent vaccine designed to provide dual protection against rotavirus and *Clostridium perfringens* alpha-toxin (CPA)-mediated disease. Leveraging an enhanced reverse genetics system, we successfully utilized the commercialized rotavirus vaccine strain LLR as a viral vector to express the key *C. perfringens* virulence factor, CPA. This strategy not only offers a path to broader protection against diarrheal disease but also establishes a versatile platform for developing vaccines against other viral-bacterial co-infections.

## INTRODUCTION

Acute infectious diarrhea poses a significant global health burden, with a complex etiology involving key pathogens such as rotavirus (RV), *Clostridium perfringens*, norovirus, and enterotoxigenic *Escherichia coli* (1, 2). Among these, RV is the primary cause of severe dehydrating diarrhea in children globally (3), while *C. perfringens* is a predominant agent of foodborne gastroenteritis, particularly in the United States (4). Crucially, both pathogens compromise intestinal barrier integrity through toxin-mediated mucosal damage and inflammation while simultaneously suppressing host immunity. This dual disruption facilitates secondary infections and exacerbates disease severity (5, 6), potentially leading to life-threatening complications such as bacteremia, sepsis, and multi-organ failure (7–9). The lack of a licensed *C. perfringens* vaccine (10), coupled with the severe outcomes of co-infection, underscores a critical unmet need and mandates the accelerated development of a bivalent prophylactic strategy.

Rotavirus remains a leading cause of mortality in children under 5, accounting for an estimated 122,000–216,000 annual deaths globally, with approximately 85% occurring in developing countries (11). Its zoonotic potential further elevates public health risks (12). Consequently, the World Health Organization (WHO) recommends the inclusion of rotavirus vaccines in all national immunization programs (13, 14). The Lanzhou lamb rotavirus vaccine (LLR), developed by the Lanzhou Institute of Biological Products, is derived from a lamb rotavirus strain (G10P[15]) attenuated through serial passage in calf kidney cells. Licensed in China for pediatric use since 2000, over 83 million doses of LLR have been distributed (National Institutes for Food and Drug Control, Vaccine Lot Release System; October 2019) (15). While efficacy data from randomized controlled trials are limited, post-marketing studies in China report vaccine effectiveness ranging from 35.0% (95% CI: 13.0–52.0) for any-severity cases to 73.3% (95% CI: 61.2–81.6) for hospitalization-required cases (16, 17). This established public health benefit, combined with its thermostability, positions LLR as an ideal mucosal vaccine platform for resource-limited regions.

*C. perfringens* comprises seven toxigenic types (A–G), with types A, C, and F being pathogenic in humans (18). These strains produce six major toxins, including alpha (CPA), beta, epsilon, iota, enterotoxin, and NetB-like, which are responsible for various human and animal diseases (10). Notably, CPA, a zinc-dependent phospholipase C produced by all *C. perfringens* types, is a critical virulence factor. It hydrolyzes membrane phospholipids, resulting in cell lysis, tissue necrosis, and systemic complications (19). Structurally, CPA consists of an N-terminal domain and a C-terminal domain (CTD) that mediate membrane binding and contain protective epitopes (20). While conventional toxoid vaccines exist, their efficacy is hindered by complex antigenicity, inconsistent immunogenicity, and hazardous requirement for native toxin production (21). A promising alternative involves targeted mutagenesis of key catalytic histidine residues (H11G/H68G/H126G), which abolishes CPA’s hemolytic activity while retaining its immunogenicity (22), thereby enabling the development of safer, precision-engineered antigens.

Here, we report the development of a novel bivalent vaccine candidate designed to confer dual protection against rotavirus and *C. perfringens* alpha-toxin. Using the licensed LLR vaccine strain as a viral vector (23), we engineered a recombinant rotavirus expressing a modified, immunogenic C-terminal domain of the *C. perfringens* alpha-toxin (CPA-CTD). Oral administration in suckling mice and intramuscular delivery in cows both elicited robust systemic antibody responses against both pathogens and passive immunity. This represents the first rotavirus-bacterial vector platform capable of providing concurrent protection, offering a significant advance in “One Health” strategies for integrated disease control.

## RESULTS

### Optimization of CPA-CTD expression in the rotavirus LLR strain using a glycine-serine-glycine-P2A linker

**Fig 1:**
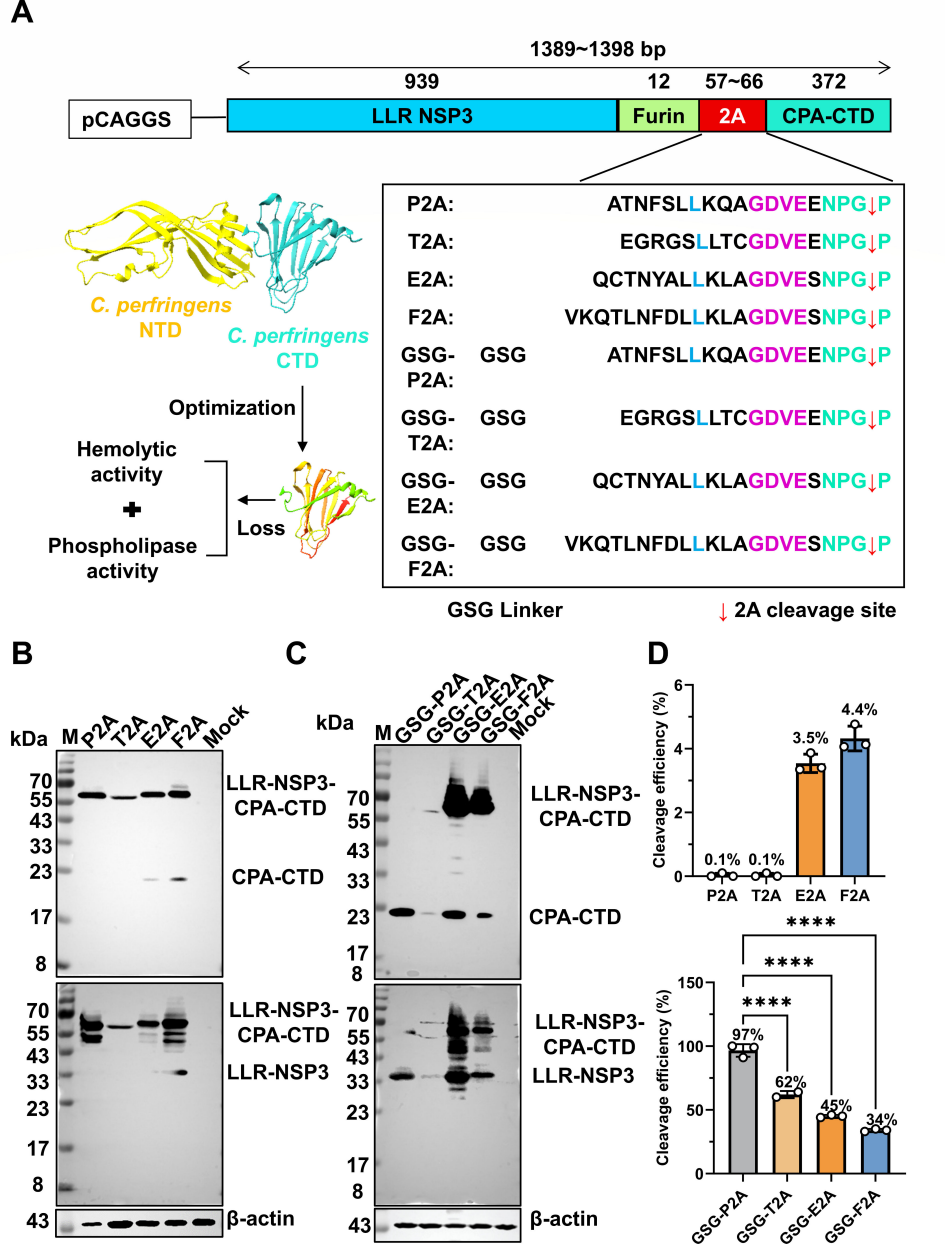
Optimization of CPA-CTD expression in rotavirus LLR strain using GSG-P2A configuration. (**A**) Schematic representation of the constructs harboring different 2A peptides for CPA-CTD expression. Eight rotavirus LLR NSP3 plasmids containing the fragment of CPA-CTD linked by the self-cleaving 2A peptides were constructed. Amino acid sequences of the 2A peptides used in this study are shown, and the conserved regions are highlighted. The cartoon illustration of the CPA monomer models was created using Swiss Model (https://swissmodel.expasy.org/, PDB ID 3am2). The N-terminal domain and the C-terminal domain are shown in dark turquoise and yellow, respectively. (**B and C**) The expression of LLR-NSP3-2A-CPA-CTD in transfected HEK 293T cells was detected by Western blot analysis. The transfected cells were processed for Western blot analysis 48 h post-transfection. The expression of the fused or cleaved proteins was assessed using mouse anti-Flag monoclonal antibody and rabbit anti-LLR-NSP3 and anti-CPA-CTD polyclonal antibodies to detect LLR-NSP3-2A-CPA-CTD and CPA-CTD, respectively. Anti-β-actin antibody was used as a loading control. (**D**) Quantitation of the cleavage efficiency of the indicated 2As. The amount of fused and cleaved proteins was measured by ImageJ software. The value was used for calculation. Data represent mean ± SD. Significance was determined by Student’s *t*-test. Statistical significance is indicated as ****P < 0.0001.

The C-terminal domain of the *C. perfringens* alpha-toxin (CPA-CTD) represents an optimal vaccine target, as it retains key immunogenicity without virulence. To exploit this, we engineered a series of constructs in which the CPA-CTD coding sequence was fused downstream of the viral NSP3 gene. Expression was mediated via a furin cleavage site followed by one of four distinct self-cleaving 2A peptides: F2A, E2A, P2A, or T2A (Fig. 1A). Transfection of these constructs into HEK 293T cells confirmed robust expression of the NSP3_LLR_-2A-CPA-CTD fusion protein for P2A, E2A, and F2A variants. However, the intrinsic 2A-mediated cleavage efficiency was suboptimal (Fig. 1B and D). To address this, we introduced a glycine-serine-glycine (GSG) spacer followed by a furin recognition site upstream of each 2A peptide. Western blot analysis revealed that the GSG linker significantly enhanced cleavage, with GSG-P2A achieving near-complete cleavage (97%), followed by GSG-T2A (62%), GSG-E2A (45%), and GSG-F2A (34%) (Fig. 1C and D). Given its superior cleavage efficiency and high antigen expression, the GSG-P2A configuration was selected as the optimal design for multigene expression in the rotavirus LLR platform.

### Generation of a recombinant RV expressing the CPA-CTD

**Fig 2:**
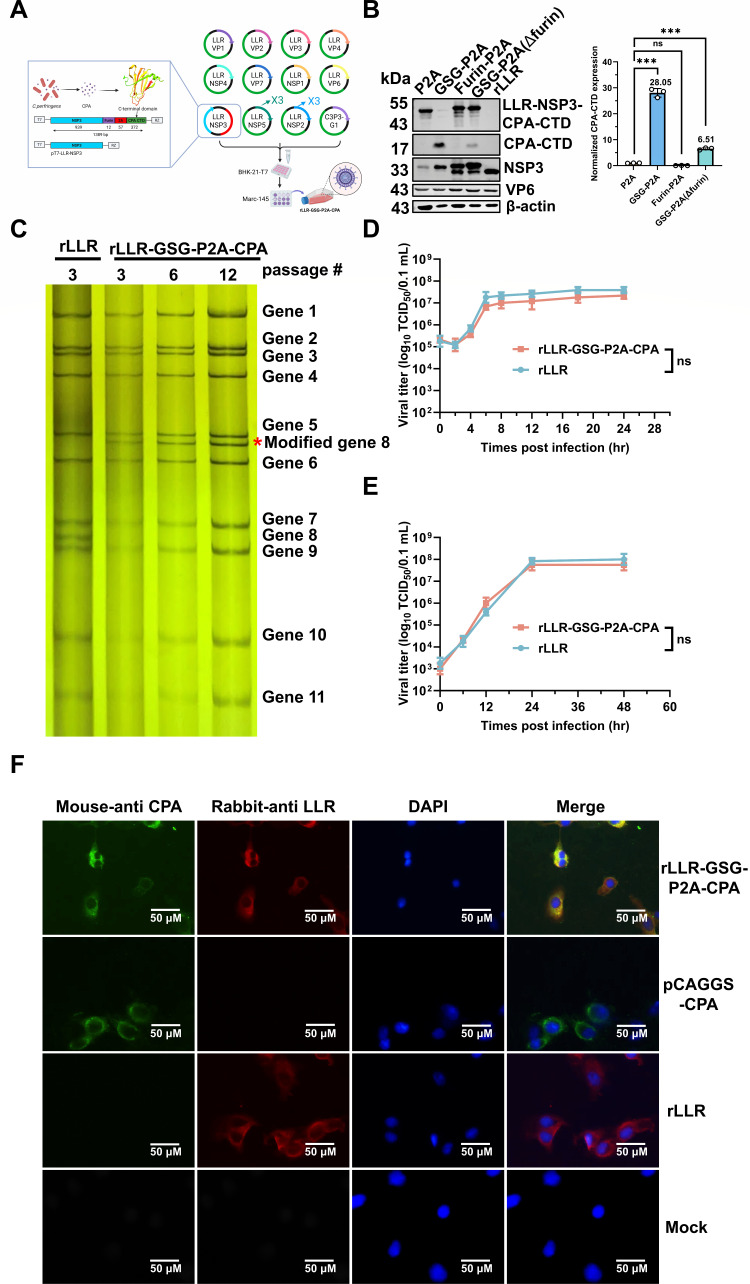
*In vitro* characterization of rLLR-GSG-P2A-CPA. (**A**) Schematic representation of the reverse genetics system for generating recombinant rotavirus expressing CPA-CTD. (**B**) Western blotting analysis of protein expression by recombinant viruses in Marc-145 cells. Marc-145 cells were infected with these recombinant viruses at an MOI of 1 and harvested at 12 h post-infection. The cells were lysed with NP40 buffer and resolved by sodium dodecyl sulfate polyacrylamide gel electrophoresis (SDS-PAGE). Protein expression of CPA-CTD, LLR-NSP3, LLR-VP6, and β-actin was detected by specific antibodies. The numbers indicate the molecular weights of the protein ladder. (**C**) Electrophoretic analysis of viral dsRNA. Viral dsRNAs were purified from rLLR and different passages of rLLR-GSG-P2A-CPA, separated by RNA-PAGE, and visualized by silver staining. The modified gene segment 8 is indicated by a red asterisk. (**D and E**) Growth kinetics of rLLR and rLLR-GSG-P2A-CPA in Marc-145 cells. Marc-145 cells were infected with either rLLR or rLLR-GSG-P2A-CPA at an MOI of 1 (**D**) or 0.01 (**E**). The samples were frozen at each indicated time point, and the virus titers were determined by a TCID_50_ assay. Representative data of two independent experiments with triplicate samples are shown as mean with standard deviation (SD). Statistical analysis was performed by Student’s *t*-test. Statistical significance is indicated as ns (*P* > 0.05) and *** (*P* < 0.001). (**F**) Immunostaining analysis of protein expression by rLLR-GSG-P2A-CPA in Marc-145 cells. Marc-145 cells were infected with the rLLR or rLLR-GSG-P2A-CPA at an MOI of 1 and fixed at 24 h post-infection, or the cells were transfected with the pCAGGS-CPA plasmid and fixed at 48 h post-transfection. The cells were stained with antibodies specific to LLR (red), CPA (green), and 4′,6-diamidino-2-phenylindole dihydrochloride (DAPI) (blue). Representative data from two independent experiments are shown. (Scale bar, 50 μm.)

To generate a recombinant rotavirus expressing the CPA-CTD, we sequentially cloned the coding sequences for a furin cleavage site, a GSG linker, the P2A, and the CPA-CTD in-frame into the pT7-LLR-NSP3 plasmid (Fig. 2A). For comparison, we constructed three control plasmids: P2A (no furin and GSG), furin-P2A (furin without GSG), and GSG-P2A(Δfurin) (GSG without furin). Using our established reverse genetics system (24), we successfully rescued four corresponding recombinant LLR strains: rLLR-GSG-P2A-CPA, rLLR-P2A-CPA, rLLR-furin-P2A-CPA, and rLLR-GSG-P2A(Δfurin)-CPA. Western blot analysis confirmed that the cleaved CPA protein expression was significantly higher in rLLR harboring GSG linker (rLLR-GSG-P2A-CPA and rLLR-GSG-P2A(Δfurin)-CPA), compared to those without it. Only rLLR-GSG-P2A-CPA produced the fully cleaved 14 kDa CPA-CTD protein. Quantification revealed a 28.05-fold higher CPA-CTD expression level in this strain compared to rLLR-P2A-CPA (Fig. 2B), underscoring the critical role of the GSG spacer in enhancing both cleavage efficiency and antigen yield. RNA-PAGE analysis of passage 3 (P3) virus stocks showed an additional 1,533 bp band, corresponding to the modified gene segment 8, migrating between segments 5 (1,611 bp) and 6 (1,356 bp). This modified segment remained genetically stable over serial passages (P3, P6, and P12) (Fig. 2C). Growth kinetics in Marc-145 cells demonstrated that rLLR-GSG-P2A-CPA replicated efficiently at both high (MOI = 1) (Fig. 2D) and low (MOI = 0.01) multiplicities of infection (Fig. 2E). Although the peak virus titer was slightly lower than that of rLLR, the difference was not statistically significant (Fig. 2D and E). Immunostaining further confirmed that 100% of cells infected with rLLR-GSG-P2A-CPA expressed the CPA-CTD antigen (Fig. 2F). Collectively, these results demonstrate the successful rescue of a recombinant rotavirus that stably maintains the modified gene segment 8 and robustly expresses the CPA-CTD immunogen in infected cells.

### Effect of CPA-CTD insertion on RV replication and pathogenesis *in vivo*

**Fig 3:**
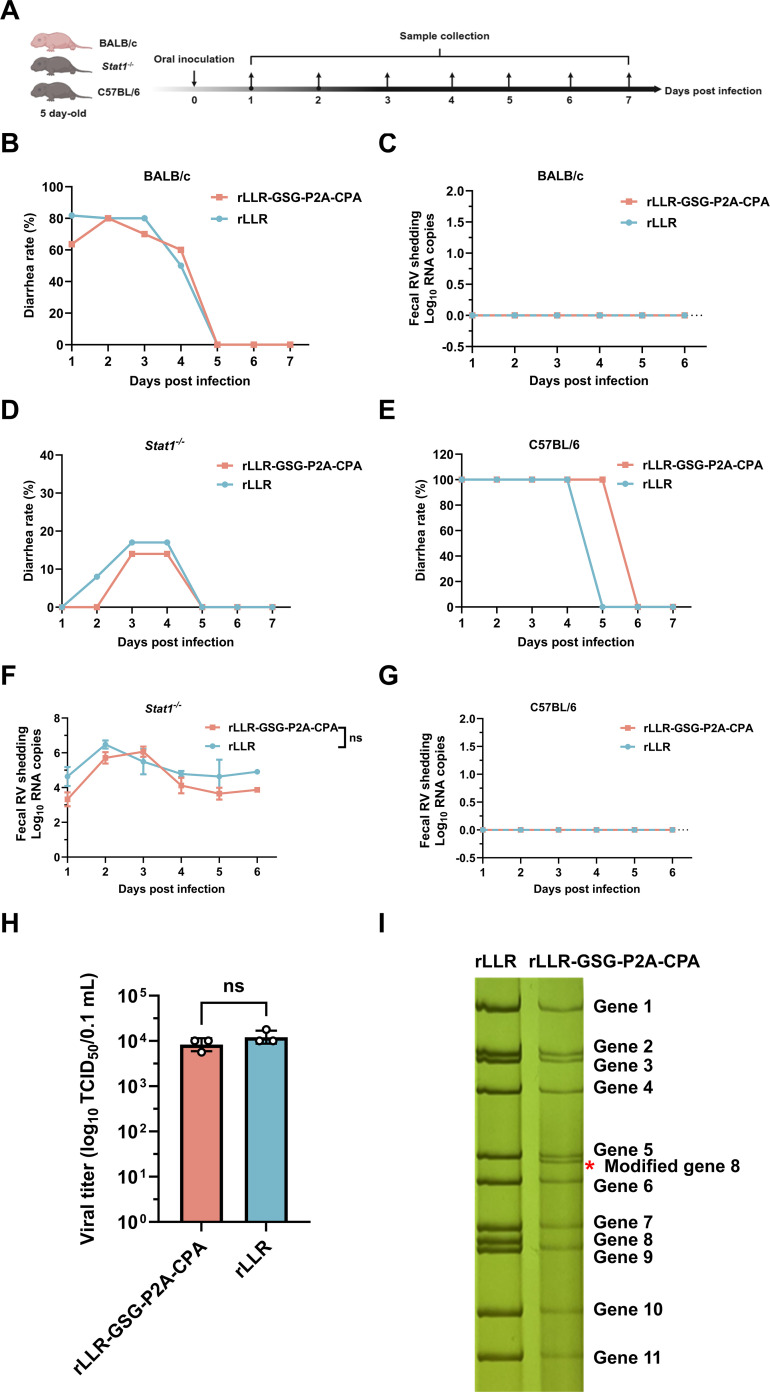
*In vivo* characterization of rLLR-GSG-P2A-CPA. (**A**) Schematic of the experimental infection design. (**B, D, and E**) Diarrhea incidence in BALB/c (**B**), Stat1^−/−^ (**D**), and C57BL/6 (**E**) neonatal mice following oral inoculation with rLLR or rLLR-GSG-P2A-CPA. Five-day-old BALB/c pups (rLLR: *n* = 7; rLLR-GSG-P2A-CPA: *n* = 7), C57BL/6 pups (rLLR: *n* = 5; rLLR-GSG-P2A-CPA: *n* = 5), and Stat1^−/−^ pups (rLLR: *n* = 13; rLLR-GSG-P2A-CPA: *n* = 14) were inoculated. Diarrhea was assessed daily for 7 days post-infection via gentle abdominal palpation; pups displaying liquid or unformed stools were scored positive. Data represent the daily percentage of pups with diarrhea. (**C, F, and G**) Fecal viral shedding kinetics in BALB/c (**C**), Stat1^−/−^ (**F**), and C57BL/6 (**G**) mice after inoculation. Rotavirus RNA in stool samples collected daily from days 1 to 6 post-infection was quantified by qPCR (Thermo Fisher Scientific). Data represent mean ± SD. Statistical analysis was performed with two-way ANOVA. Statistical significance is indicated as ns, not significant. (**H**) Detection of rotavirus titer in fecal samples from Stat1^−/−^ mice. (**I**) Electrophoretic analysis of viral dsRNA in fecal samples. Fecal samples were collected from Stat1^−/−^ mice at 6 dpi and then amplified the virus in Marc-145 cells until the appearance of evident CPE. Viral dsRNAs were purified and separated by RNA-PAGE and visualized by silver staining. The modified gene segment 8 is indicated by a red asterisk.

To evaluate the impact of CPA-CTD insertion on viral replication and safety, we orally inoculated 5-day-old BALB/c mouse pups with either rLLR-GSG-P2A-CPA or wild-type rLLR (5 × 10⁷ TCID_50_) and monitored diarrhea incidence and fecal viral shedding for 7 days (Fig. 3A). Both viruses induced diarrhea during the first 4 days post-inoculation (Fig. 3B), yet viral RNA remained undetectable by quantitative real-time PCR (qPCR) in stool samples from either group (Fig. 3C). Since rotavirus-induced diarrhea is linked to innate immune sensing in intestinal epithelial cells (25), we next assessed the viral replication in 5-day-old Stat1^−/−^ mice, which exhibit impaired interferon signaling. Consistent with prior reports (25, 26), both rLLR and rLLR-GSG-P2A-CPA caused significantly less diarrhea in Stat1^−/−^ mice compared to immunocompetent C57BL/6 mice (Fig. 3D and E). Notably, low-level viral shedding was detected in fecal samples from Stat1^−/−^ mice (days 1–6 post-inoculation), with no significant difference between viruses, whereas no viral RNA was detected in wild-type C57BL/6 mice (Fig. 3F and G). This trend was mirrored in infectious virus titers. Only fecal samples from Stat1^−/−^ mice at 2 dpi yielded detectable titers, with rLLR-GSG-P2A-CPA showing 10^3.9^ TCID_50_/0.1 mL and rLLR yielding 10^4.1^ TCID_50_/0.1 mL (Fig. 3H). Subsequent sequencing of the virus isolated from Stat1^−/−^ mouse feces at 6 dpi confirmed that rLLR-GSG-P2A-CPA retained the CPA-CTD coding sequence (Fig. 3I). Together, these results demonstrate that insertion of the CPA-CTD antigen does not alter the replication kinetics profile of the LLR vaccine backbone *in vivo*.

### Serum IgG and IgA responses in BALB/c suckling mice following vaccination with rLLR-GSG-P2A-CPA

**Fig 4:**
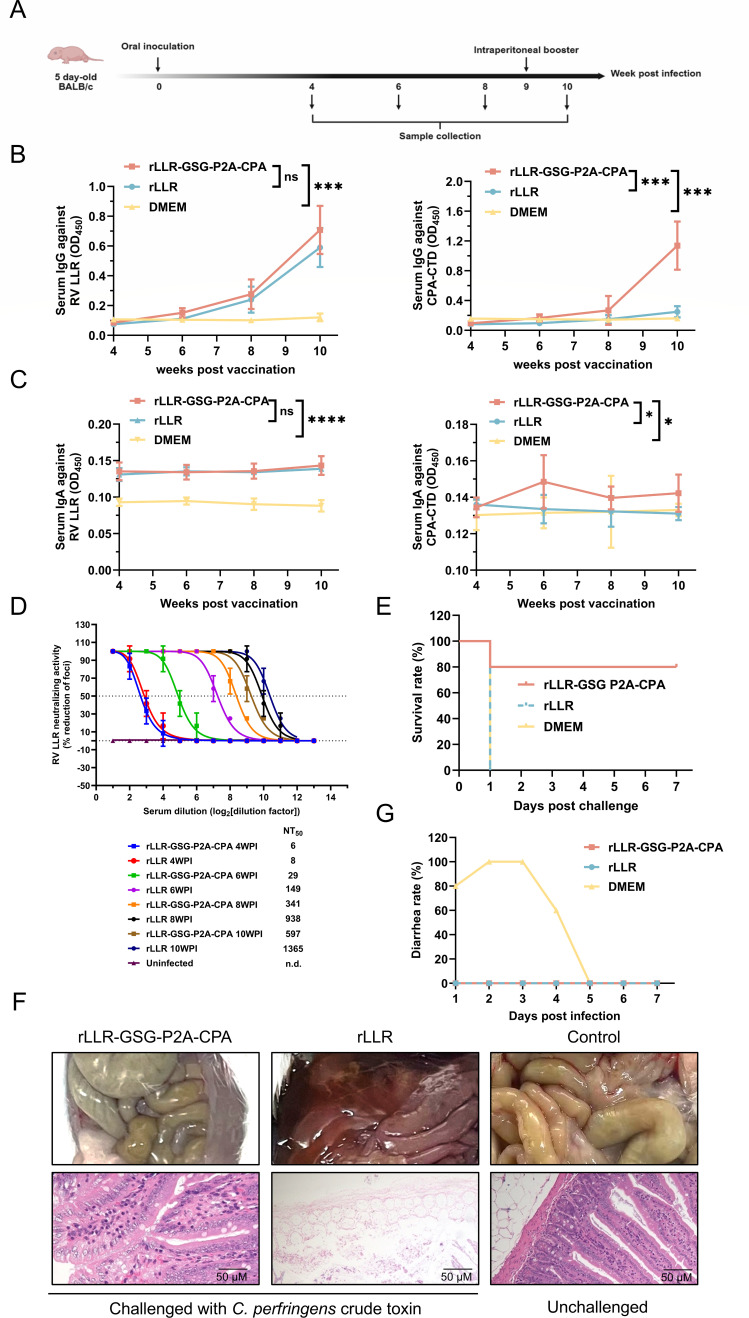
Systemic immune responses and protective efficacy of rLLR-GSG-P2A-CPA in BALB/c mice. (**A**) Immunization and sampling scheme. Five-day-old BALB/c pups received oral inoculation (5 × 10^7^ TCID_50_) of rLLR or rLLR-GSG-P2A-CPA at week 0, followed by intraperitoneal booster immunization (same dose) at 9 weeks post-inoculation (WPI). Sera were collected at the indicated time points. (**B**) Kinetics of serum IgG responses against rotavirus LLR and CPA-CTD. Antibody levels (OD_450_) were measured by ELISA at 1:100 serum dilution. Data represent mean ± SD. Statistical analysis was performed using two-way ANOVA (ns, not significant; ***P < 0.001). (**C**) Kinetics of serum IgA responses against rotavirus LLR and CPA-CTD. Antibody levels (OD_450_) were measured by ELISA at 1:100 serum dilution. Data represent mean ± SD. Statistical analysis was performed using two-way ANOVA (ns, not significant; *P < 0.05; ****P < 0.0001). (**D**) Neutralizing antibody titers against rotavirus LLR. Titers were determined by focus reduction assay (focus forming units, FFU) after serum-virus incubation (37°C, 1 h) in Marc-145 cells. Data show mean percent focus reduction ± SD. n.d.: not detected (<1:2). (**E**) Survival rates following lethal challenge with *C. perfringens* crude toxin at 10 WPI. (**F**) Representative intestinal pathology: gross examination (top) and histology (hematoxylin and eosin staining, 400× magnification; bottom) post-challenge. (**G**) Diarrhea incidence in BALB/c neonatal mice following rotavirus infection. At the seventh week after immunization with rLLR-GSG-P2A-CPA, immunized female mice were mated with male BALB/c mice. Post-delivery, pups acquired maternal antibodies through suckling. At 5 days old, the pups were orally challenged with wild-type LLR rotavirus. Diarrhea was assessed daily for 7 days post-infection via gentle abdominal palpation; pups displaying liquid or unformed stools were scored positive. Data represent the daily percentage of pups with diarrhea.

To evaluate whether rLLR-GSG-P2A-CPA induces systemic humoral immunity against rotavirus LLR and CPA-CTD in neonatal mice, 5-day-old BALB/c pups were orally inoculated with 5 × 10^7^ TCID_50_ of either rLLR-GSG-P2A-CPA or rLLR. Serum was collected at 4, 6, and 8 weeks post-inoculation (WPI), followed by an intraperitoneal booster at 9 WPI and terminal collection at 10 WPI (Fig. 4A). Longitudinal analysis revealed that rLLR-GSG-P2A-CPA elicited robust IgG responses against both rotavirus LLR and CPA-CTD. Anti-rotavirus LLR IgG levels increased progressively, reaching a mean OD_450_ of 0.7 by 10 WPI, with no significant difference between the rLLR-GSG-P2A-CPA and rLLR control groups at any time point (*P* > 0.05). Notably, all mice in the rLLR-GSG-P2A-CPA group seroconverted to CPA-CTD by 6 WPI, and the booster significantly enhanced this response (Fig. 4B). Serum IgA against rotavirus LLR was detectable at 4 WPI and increased slightly after boosting. Anti-CPA-CTD IgA was also detected, emerging at 6 WPI (Fig. 4C). These results demonstrate that oral immunization with rLLR-GSG-P2A-CPA induces systemic antibody responses against both antigens and that a parenteral booster effectively amplifies this humoral immunity.

We next assessed the neutralizing activity of immune sera against rotavirus LLR and *Clostridium perfringens* alpha-toxin. Sera from rLLR-GSG-P2A-CPA-immunized mice contained neutralizing antibodies against rotavirus LLR, with geometric mean titers of 1:341 at 8 WPI rising to 1:597 post-booster. These titers were moderately lower than those in the rLLR control group (1:938 at 8 WPI and 1:1,365 at 10 WPI; Fig. 4D). To evaluate protection against *Clostridium perfringens* alpha-toxin, mice were challenged with a lethal dose of crude toxin 1 week post-booster. The survival rate was monitored for 7 days post-challenge. The rLLR-GSG-P2A-CPA group exhibited an 80% survival rate (4/5 mice) (Fig. 4E), with one mouse showing only mild, transient symptoms. In stark contrast, unvaccinated (Dulbecco’s modified Eagle medium [DMEM]) controls developed severe illness within 12 h post-challenge and succumbed by 24 h (*P* = 0.0062). Necropsy revealed severe intestinal damage, including mucosal destruction and villi disintegration in unvaccinated challenged mice, whereas the intestinal architecture of vaccinated mice was preserved, comparable to unchallenged controls (Fig. 4F). To further assess protection against rotavirus, a maternal immunization model was employed. Female mice immunized with rLLR-GSG-P2A-CPA were mated 7 weeks post-immunization. Their suckling pups, having acquired maternal antibodies, were orally challenged with wild-type LLR rotavirus at 5 days of age. In the unvaccinated control group, 80% of pups (4/5) developed severe diarrhea by day 1 post-infection, and 100% (5/5) were affected by day 2. In contrast, no diarrheal symptoms were observed in pups from either the rLLR-GSG-P2A-CPA or the rLLR-immunized groups (0/5) at any time point (Fig. 4G). Collectively, these data demonstrate that rLLR-GSG-P2A-CPA elicits neutralizing antibodies against rotavirus LLR and confers significant protection against lethal challenge with both *C. perfringens* alpha-toxin and rotavirus.

### rLLR-GSG-P2A-CPA elicits robust systemic and maternal immunity in cows

To identify an optimal vaccination strategy, we compared the immunogenicity of live versus inactivated rLLR-GSG-P2A-CPA in mice via intramuscular injection (Fig. S1A). The live vaccine elicited a significant RV LLR-specific IgG response, with titers escalating after a booster. Notably, it also triggered CPA-CTD-specific seroconversion within 7 days post-vaccination, a response not observed with the inactivated vaccine (Fig. S1B). While the inactivated vaccine generated higher neutralizing antibody titers against RV LLR (1:2,901 versus 1:1,706), only the live vaccine conferred significant protection, yielding a 40% survival rate against a lethal *C. perfringens* crude toxin challenge, compared to 100% mortality in both the inactivated vaccine and control groups (Fig. S1C and D). These results confirm the flexibility of our vaccine platform, demonstrating that robust dual-antigen immunity in mice can be achieved not only through oral delivery but also via intramuscular injection.

**Fig 5:**
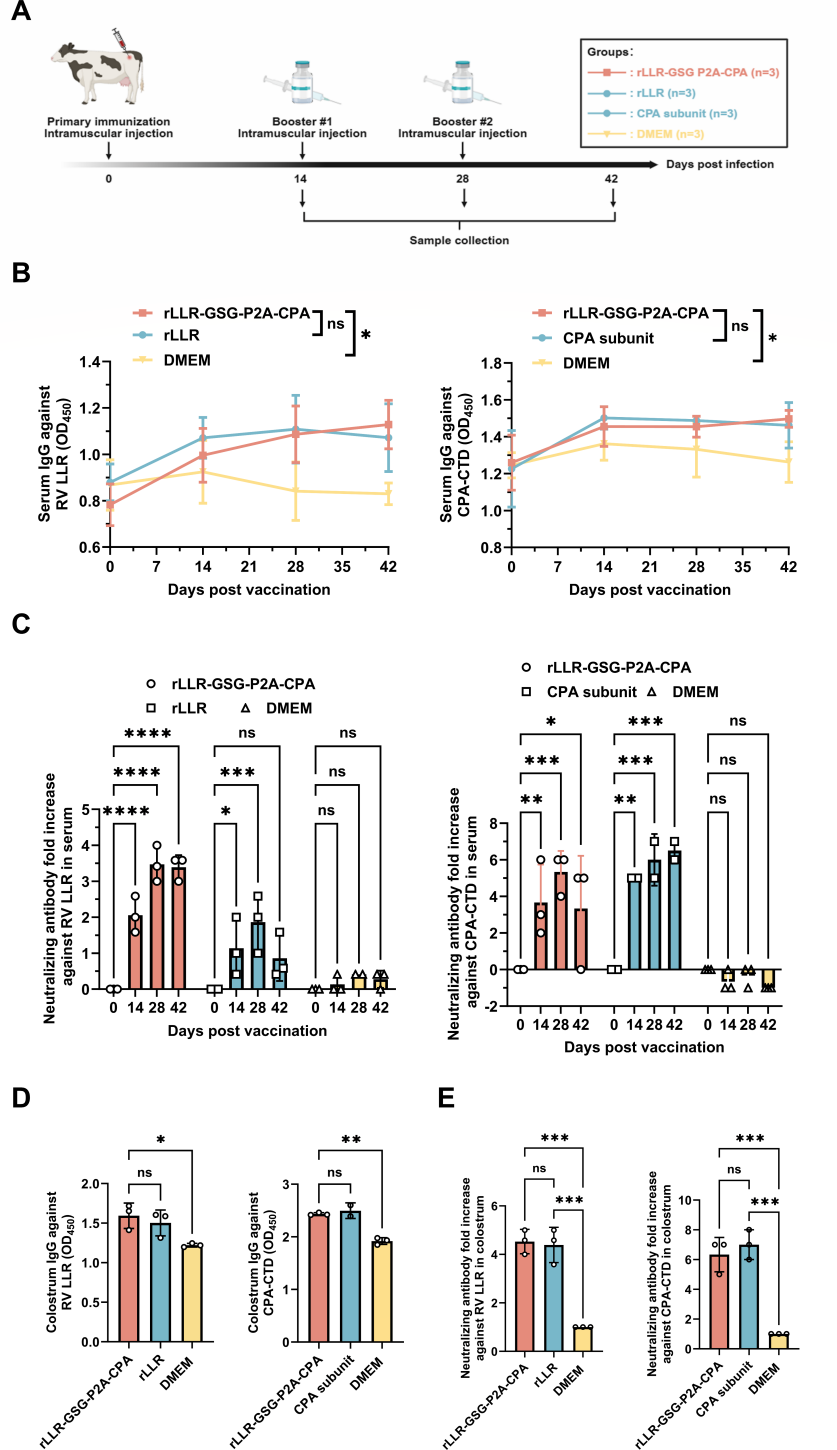
rLLR-GSG-P2A-CPA immunization induces robust specific antibody responses in the serum and colostrum of cows. (**A**) Immunization and sampling scheme. Cows were immunized intramuscularly with infectious rLLR-GSG-P2A-CPA (5 × 10^8^ TCID_50_) mixed with an oil adjuvant, ISA 15A VG, at a ratio of 85:15 (vol/vol). For the CPA subunit vaccine, cows were immunized intramuscularly with 200 µg of CPA protein mixed with an oil adjuvant, ISA 15A VG, at a ratio of 85:15 (vol/vol), and boosted with the same dose 2 and 4 weeks later. Sera were collected at the indicated time points. (**B**) Kinetics of serum IgG responses against rotavirus LLR and CPA-CTD. Antibody levels (OD_450_) were measured by ELISA at 1:100 serum dilution. Data represent mean ± SD. Statistical analysis was performed using two-way ANOVA (ns, not significant; *P < 0.05). (**C**) Fold increase of neutralizing antibody titers against rotavirus LLR and CPA-CTD in serum compared to pre-inoculation.Statistical analysis was performed using two-way ANOVA (ns, not significant; *P < 0.05; **P < 0.01; ***P < 0.001; ****P < 0.0001). (**D**) Colostrum IgG responses against rotavirus LLR and CPA-CTD. Colostrum from pregnant cows on the first day after calving was collected, and antibody levels (OD_450_) were measured by ELISA. Data represent mean ± SD. Statistical analysis was performed using two-way ANOVA (ns, not significant; *P < 0.05; **P < 0.01). (**E**) Fold increase of neutralizing antibody titers against rotavirus LLR and CPA-CTD in colostrum compared to the control. Data represent mean ± SD. Statistical analysis was performed using two-way ANOVA (ns, not significant; ***P < 0.001).

Given that oral administration of bovine colostrum containing rotavirus-specific antibodies can prevent rotavirus infection (27, 28), we next evaluated the vaccine in a bovine maternal immunization model. Cows received prime/boost intramuscular immunizations prepartum (days 0, 14, and 28) with rLLR-GSG-P2A-CPA (5 × 10⁸ TCID_50_), formulated with ISA 15A VG adjuvant (85:15 vol/vol) (Fig. 5A). Serum and colostrum were collected for analysis. ELISA revealed strong serum IgG responses against both rotavirus LLR and CPA-CTD, with titers increasing after each immunization (Fig. 5B). A similarly robust IgG response was detected in colostrum, confirming efficient antibody transfer (Fig. 5D). Neutralization assays demonstrated that 28 days post-vaccination serum from immunized cows showed a 3.47-fold increase in anti-RV and a 5.33-fold increase in anti-CPA-CTD neutralizing titers compared to pre-vaccination levels (Fig. 5C). Colostrum from vaccinated cows also contained high levels of neutralizing antibodies, exhibiting 4.53-fold and 6.33-fold increases against rotavirus LLR and CPA-CTD, respectively, compared to controls (Fig. 5E). Together, these data demonstrate that the live rLLR-GSG-P2A-CPA vaccine elicits a potent systemic humoral response in cows and promotes the efficient transfer of neutralizing antibodies into colostrum. This supports the potential for conferring passive immunity against both rotavirus and *C. perfringens* alpha-toxin to infants through bovine colostrum ingestion.

## DISCUSSION

Diarrheal diseases remain a leading cause of global morbidity and mortality, particularly in children under 5 in resource-limited settings (4). While often attributed to a single pathogen, clinical studies increasingly reveal that polymicrobial infections, involving synergistic interactions between viruses and bacteria, are frequent and associated with more severe disease outcomes. The co-occurrence of enteric viruses such as rotavirus with bacterial pathogens such as *C. perfringens* represents a significant threat to human and animal health that is inadequately addressed by current monovalent vaccination strategies (29). This study demonstrates the feasibility of a novel bivalent vaccine platform designed to concurrently target these synergistic pathogens, thereby addressing a critical gap in our preventive strategies.

While the P2A peptide is widely used in viral vectors to separate heterologous antigens, we found the native P2A sequence mediated only partial cleavage of an LLR-NSP3-CPA-CTD fusion protein. Incorporating a GSG linker upstream of the 2A sequence significantly improved cleavage efficiency, with the GSG-P2A configuration achieving near-complete processing (97%). This is consistent with studies indicating that GSG linkers reduce steric hindrance and enhance conformational flexibility (30–33). This optimized design enables high-yield expression of free CPA-CTD without compromising viral protein integrity, establishing a reliable co-expression system for the rotavirus backbone.

Using this design, we generated a recombinant rotavirus (rLLR-GSG-P2A-CPA) that stably expresses CPA-CTD. The virus retained the modified gene segment 8 over serial passages and produced high levels of the 14 kDa CPA-CTD in infected cells. Importantly, antigenic insertion did not affect viral replication kinetics or attenuation *in vitro* or *in vivo*. In both immunocompetent and Stat1^−/−^ neonatal mice, the recombinant virus exhibited fecal shedding and diarrheal profiles indistinguishable from the parental rLLR strain, confirming its genetic stability.

Immunization with rLLR-GSG-P2A-CPA induced robust systemic IgG against both rotavirus LLR and CPA-CTD in neonatal mice. Although rotavirus-neutralizing titers were moderately lower than those induced by rLLR alone, the difference was not statistically significant (*P* > 0.05). Maternal antibodies generated by either rLLR-GSG-P2A-CPA or rLLR effectively protected suckling mice from rotavirus challenge, conferring 100% protection against diarrhea. Moreover, the vaccine also elicited significant protection against lethal *C. perfringens* crude toxin challenge, with 80% survival and preserved intestinal architecture, confirming the functional efficacy of CPA-CTD-specific immunity. The platform demonstrated flexibility in immunization route: while oral delivery elicited stronger responses in mice, intramuscular administration of the live vaccine also induced seroconversion and protection, albeit to a lesser degree, highlighting the adaptability of the LLR vector to different vaccination strategies.

The pathogenicity of *C. perfringens* is mediated primarily by its secreted exotoxins, with CPA being a key virulence factor in gas gangrene and necrotizing enteritis (34, 35). The observed protection likely results from high-titer neutralizing antibodies targeting CPA-CTD (22). Rather than inhibiting enzymatic activity directly, these antibodies are thought to act through steric hindrance or conformational interference, preventing toxin binding to host cell membranes (36). This upstream interception aligns with the established paradigm of toxoid-based vaccines, such as those for tetanus and diphtheria, where the protective mechanism is also through targeting key functional domains of the toxin to block toxin-host cell interactions and thus prevent disease (37–39).

In cows, rLLR-GSG-P2A-CPA vaccination induced robust serum and colostrum antibody responses with significant neutralizing activity against both rotavirus and CPA-CTD. The efficient transfer of antibodies into colostrum supports the potential of maternal vaccination to confer passive immunity to neonates. However, pre-existing immunity to rotavirus may limit the virus replication and heterologous antigen immunogenicity (40). Although cows with low baseline rotavirus-neutralizing titers (<1:128) were selected, higher pre-existing antibody levels correlated with reduced CPA-CTD immunogenicity. Further optimization, such as heterologous prime–boost regimens or neonatal immunization strategies, may help overcome this limitation.

Mouse experiments revealed that oral immunization elicited markedly superior protection compared to intramuscular delivery, underscoring the importance of route in shaping immune outcomes. For enteric pathogens like rotavirus, immune defenses mounted at the intestinal mucosa are most effective. In neonatal calves or infants, oral delivery should therefore be prioritized to induce robust mucosal immunity. However, the immature gastrointestinal tract in early life presents a challenge, as orally administered live vaccines can be degraded by pepsin in a strain-dependent manner (41). Consequently, for adult cattle, the intramuscular route remains a reliable alternative, ensuring consistent antigen delivery and systemic immune activation.

In conclusion, we have developed a novel bivalent rotavirus-vectored vaccine that exploits rotavirus’s enteric tropism to effectively co-express CPA-CTD, offering protection against viral-bacterial coinfections. Built on the proven rotavirus LLR backbone and optimized for antigen expression, this platform represents a promising strategy against polymicrobial enteric diseases and enhances pandemic preparedness through single-dose prophylaxis.

## MATERIALS AND METHODS

### Cells and viruses

Baby hamster kidney cell line, BHK-T7, constitutively expressing T7 RNA polymerase, was cultured in DMEM (Sigma-Aldrich, cat. #D6429) supplemented with 10% fetal bovine serum (PAA, cat. #A-005N) (complete medium). Monkey kidney cell lines Marc-145 were cultured in complete MEM. The LLR vaccine strain was preserved in our laboratory (23). The viruses were rescued as previously described (24) and pretreated with 10 μg/mL trypsin (type IX, from porcine pancreas, Sigma-Aldrich, cat. #T0303) for propagation in Marc-145 cells in the incomplete medium (DMEM supplemented with 5 μg/mL trypsin).

### Recombinant proteins and antibodies

Recombinant CPA was generated as previously described (21). The antibodies used in this study are as follows: anti-LLR rabbit antiserum, anti-NSP3 mouse antiserum, and anti-CPA mouse antiserum, all of which were generated in our laboratory. Commercially obtained antibodies included anti-β-actin mouse monoclonal antibody (Sigma-Aldrich, #A2228), DYKDDDDK tag monoclonal antibody (Proteintech, #8H6A10), goat anti-mouse IgA (Immunoway, RS030211), and the following secondary antibodies from Thermo Scientific: goat anti-mouse IgG (#31431), goat anti-rabbit IgG (#31460), goat anti-mouse IgG Alexa Fluor 488 (#A-11029), and goat anti-rabbit IgG Alexa Fluor 594 (#A-11008).

### Plasmid construction

The expression vector pCAGGS-LLR-NSP3-2A-CPA-CTD was constructed as follows. The genes encoding the NSP3 protein of rotavirus LLR strain and the CPA-CTD were individually amplified by PCR. These amplicons were sequentially inserted into the eukaryotic expression vector pCAGGS using the In-Fusion HD Cloning Plus (Takara, cat. #638911). To enable expression of CPA-CTD, four distinct self-cleaving 2A peptides were fused to the C-terminus of the NSP3 open reading frame (ORF), thereby linking it upstream of the CPA-CTD ORF. The amino acid sequences of the 2A peptide are provided in Fig. 1. To improve cleavage efficiency, a furin cleavage site followed by a flexible linker (Gly-Ser-Gly, GSG) was incorporated upstream of each 2A sequence. To construct the plasmid harboring CPA-CTD in rotavirus gene 8 (pT7-LLR-NSP3-GSG-2A-CPA-CTD), the CPA-CTD fragment from *C. perfringens* type A strain CVCC C57-8 was amplified using PrimeStar HS DNA polymerase (Takara Bio, cat. #r010a). The amplicon was subsequently used to replace the ZsGreen coding sequence in the previously established plasmid pT7-LLR-NSP3-2A-ZsGreen, constructed via the In-Fusion HD Cloning Plus (24).

### Reverse genetics

Recombinant viruses were rescued following the previously described reverse genetics protocol with minor modifications (24). Briefly, monolayers of BHK-T7 cells (5 × 10^5^ cells) in 12-well plates were co-transfected with plasmids using 13.5 μL of Lipofectamine 3000 (Thermo Fisher Scientific, cat. #L3000015) transfection reagent per microgram of plasmid DNA, as follows: 0.6 μg of each rescue plasmid—pT7-LLR-VP1, pT7-LLR-VP2, pT7-LLR-VP3, pT7-LLR-VP4, pT7-LLR-VP6, pT7-LLR-VP7, pT7-LLR-NSP1, pT7-LLR-NSP3 (pT7-LLR-NSP3-GSG-2A-CPA-CTD), and pT7-LLR-NSP4—and 1.8 μg of pT7-LLR-NSP2 and pT7-LLR-NSP5. After 48 h of incubation in serum-free DMEM, Marc-145 cells (2 × 10^5^ cells) were added to the transfected cells and cocultured for 48 h in serum-free DMEM supplemented with trypsin (1 μg/mL). After incubation, the cells were frozen/thawed and passaged in Marc-145 cells, and the rescued virus was propagated with Marc-145 cells in DMEM in the presence of 0.5 μg/mL trypsin.

### RNA-PAGE analysis of viral dsRNA and genetic stability

rLLR or rLLR-GSG-P2A-CPA was passaged in Marc-145 cells. Viral genomic dsRNAs were extracted from passage 3 stock of rLLR, passages 3, 6, and 12 stocks of rLLR-GSG-P2A-CPA with QIAamp Viral RNA Mini Kit (Qiagen, cat. #52904) according to the manufacturer’s instructions. The dsRNAs were electrophoresed in 10% polyacrylamide gels for 16 h at 20 mA at room temperature, followed by silver staining to determine the genomic dsRNA migration profiles.

### Growth kinetics

A monolayer of Marc-145 cells was infected with rLLR or rLLR-GSG-P2A-CPA viruses at an MOI of 1 or 0.01. After adsorption for 1 h at 37°C, cells were washed with phosphate-buffered saline (PBS) twice and cultured with DMEM in the presence of 5 μg/mL of trypsin. The cells were frozen at individual time points. Virus titer (TCID_50_) was determined using Marc-145 cells. Data are expressed as the mean ± standard deviation (SD) of triplicate samples.

### Immunostaining

Marc-145 cells were infected with rLLR or rLLR-GSG-P2A-CPA at an MOI of 1 and cultured for 24 h. The HEK 293-T cells and Marc-145 cells were fixed with 4% paraformaldehyde for 15 min, 100 μL/well, and permeabilized with PBS containing 0.2% Triton-X for 15 min, 100 μL/well, and then washed with PBS one time. Block the cells with 10% goat serum made in 1% BSA in PBS for 1 h, 200 μL/well. The cells were incubated with primary antibody mouse anti-CPA (dilution 1:1,000) and rabbit anti-LLR (dilution 1:1,000) at 37°C for 1 h, followed by Alexa 488 conjugated anti-mouse IgG (dilution 1:1,000) and Alexa 594 conjugated anti-rabbit IgG (dilution 1:1,000) at 37°C for 1 h. The nuclei were stained with DAPI (Sigma-Aldrich, cat. #D9542). The images were acquired using an inverted fluorescence microscope (AMG, Denver, CO, USA).

### Western blotting

HEK 293-T cells were transfected with X2A or GSG-X2A (X represents P, T, E, or F, corresponding to the specific 2A peptide used, 500 ng/well) by PEI and cultured for 48 h. Marc-145 cells were infected with the recombinant viruses at an MOI of 1 and cultured for 12 h. At 12 h post-infection, the cells were lysed with NP40 buffer supplemented with phenylmethylsulfonyl fluoride (Solarbio, cat. #P0100) and boiled at 95°C for 5 min. The proteins were resolved with 12% SDS polyacrylamide gel and analyzed by antibody as described using the following antibodies: DYKDDDDK tag monoclonal antibody, mouse anti-CPA, rabbit anti-LLR, mouse anti-NSP3, or anti-β-actin mouse monoclonal antibody. Secondary antibodies were goat anti-mouse IgG or goat anti-rabbit IgG. Protein expression was visualized with Clarity ECL substrate and acquired by E-Blot Touch Imager (e-BLOT). ImageJ was used to quantify Western blot band intensities via densitometry.

### Animal experiment

BALB/c and C57BL/6 mice were purchased from Liaoning Changsheng Biotechnology Co., and the Stat1^−/−^ mice (Jax #012606, B6.129S(Cg)-Stat1tm1Dlv/J) were obtained from the Jackson Laboratory (Bar Harbor, ME, USA). To assess diarrhea rate and fecal rotavirus shedding, 5-day-old BALB/c, C57BL/6, or Stat1^−/−^ pups were orally inoculated with either rLLR or rLLR-GSG-P2A-CPA (5 × 10^7^ TCID_50_/pup) and monitored for diarrhea following gentle abdominal pressure for 7 days. According to previously established criteria (42), liquid or unformed stools were considered diarrhea positive. Stool samples were collected in 40 μL of PBS and stored at −80°C until use. To evaluate serum antibody titers against rotavirus and CPA, 5-day-old BALB/c pups (*n* = 5) were orally inoculated with rLLR-GSG-P2A-CPA (5 × 10^7^ TCID_50_/pup) and intraperitoneally injected with rLLR-GSG-P2A-CPA (5 × 10^7^ TCID_50_/pup) at 9 weeks post-inoculation (WPI). Blood samples were collected at 4, 6, 8, and 10 WPI. To establish an optimal vaccine platform, infectious or inactivated rLLR-GSG-P2A-CPA (5 × 10^7^ TCID_50_) was emulsified with ISA 15A VG at a ratio of 85:15 (vol/vol). Six-week-old female BALB/c mice (*n* = 5) were immunized via intramuscular injection on day 0 (prime) and day 14 (boost). Blood samples were collected at 7, 14, 21, and 28 DPI. To evaluate the immunogenicity of rLLR-GSG-P2A-CPA in cows, the rLLR-GSG-P2A-CPA (5 × 10^8^ TCID_50_) was mixed with ISA 15A VG and administered via intramuscular injection on day 0 (prime), day 14 (first boost), and day 28 (second boost). Blood samples were collected at 14, 28, and 42 DPI. All blood samples were centrifuged at 2,300 × *g* for 5 min, and the supernatant was collected as serum. Colostrum was obtained from pregnant cows on the first day after calving, centrifuged at 10,000 × *g* for 10 min, and the middle clear layer was filtered through a 0.22-μm membrane for subsequent analysis.

### Quantitative real-time PCR for detecting fecal rotavirus antigens

Rotavirus fecal shedding was determined by quantitative real-time PCR (qPCR) (43). Briefly, RNA was extracted from fecal samples according to the manufacturer’s instructions for QIAamp Viral RNA Mini Kit. The obtained nucleic acids were stored at −80°C until needed. qPCR was performed using the HiScript II U^+^ One Step qRT-PCR Probe Kit (Vazyme, cat. #Q223-01). The total volume of the PCR system was set as 10 µL, including 2× One Step U^+^ MIX, One Step U^+^ Enzyme MIX, and 50× ROX Reference Dye 2, and the final concentration for the primer and probe was both set as 10 µM. Amplification was carried out using the following program: 55°C for 15 min, 95°C for 30 s, 45 cycles of 95°C for 10 s, and the annealing temperature was optimized between 54°C and 60°C. Amplification was performed using Quant Studio Design & Analysis Software. Signals were automatically collected at the end of each cycle.

### ELISA for detection of serum or colostrum IgG and IgA against rotavirus and CPA-CTD

To detect serum or colostrum IgG and IgA against LLR or CPA-CTD, an indirect ELISA was performed. The ELISA plate was coated with LLR (dilution 1:4) or CPA-CTD (200 ng/well) in 0.05 M bicarbonate buffer (pH 9.6), incubated overnight at 4°C and blocked with PBS containing 2% BSA. After washing, the plates were incubated with serially diluted sera or colostrum at 37°C for 1 h. The amount of serum or colostrum IgG was detected by HRP-conjugated anti-mouse IgG (or IgA) or anti-bovine IgG (dilution 1:20,000) and visualized with a peroxidase substrate. The OD_450_ was detected by a microplate reader, ELx800.

### Rotavirus neutralizing assay

For the neutralization assay, sera were heat-inactivated at 56°C for 30 min. Following inactivation, sera or colostrum samples were then diluted in twofold serial dilutions (1:4 to 1:2,048) in media (MEM). Virus equivalent to 100 TCID_50_ was added to each well. Plates containing virus and serum or colostrum dilutions were incubated at 37°C for 1 h. Marc-145 cells were cultured for 48 h, washed twice in PBS, and then virus and serum or colostrum dilutions were added to the cell plates. After 1 h of incubation, the cells were washed with PBS and incubated with serum-free DMEM containing 5 μg/mL trypsin for 5 days. Indirect immunofluorescence assay (IFA) was performed, as described previously (26), to determine rotavirus LLR-infected Marc-145 cells. Rotavirus LLR neutralizing activities were quantified as the percentage reduction in the number of foci compared to rotavirus LLR alone. The neutralizing antibody titer was calculated as the ≥50% focus reduction neutralizing test titer (NT_50_).

### CPA challenge and histologic examination

The *C. perfringens* alpha-toxin challenge method used in this study was established in our laboratory (44). Briefly, at 10 WPI, mice from the rLLR-GSG-P2A-CPA, rLLR, and DMEM groups were intraperitoneally administered 1 LD_100_ of *C. perfringens* crude toxin, while an unchallenged control group was included. Clinical signs were monitored daily for 1 week after challenge, and survival rates were recorded. All intestinal tissue samples were collected when the mice were moribund or euthanized at 7 days post-challenge. A section of intestinal tissue was preserved in formalin for histopathological analysis. Tissues were fixed in 10% formalin, embedded in paraffin, and sectioned into 5 μm slices. Sections were mounted onto glass slides and stained with hematoxylin and eosin. The histological and morphological alterations of intestinal tissues were observed and photographed under a light microscope and independently evaluated by two researchers in a blinded manner.

### CPA neutralizing assay

A mouse neutralization test was performed to evaluate the serum-neutralizing activity of the immunized cows, as previously described (45). Sera were heat-inactivated at 56°C for 30 min. Serum or colostrum samples were then diluted in twofold serial dilutions in PBS. Each dilution was mixed with PBS containing crude alpha-toxin at 1 LD_100_ per mouse and incubated at 37°C for 1 h. The mixtures were then intraperitoneally injected into groups of four unimmunized healthy mice each. The titers of neutralizing antibodies were determined and are expressed as the highest dilution of 0.1 mL of serum that could neutralize 1 LD_100_ unit of crude toxin, resulting in a 100% survival rate in mice.

### Statistical analysis

Except for special needs, the data were analyzed by Student’s *t*-test or two-way ANOVA using the GraphPad Prism 10.0 software (GraphPad, La Jolla, CA, USA) and were presented as mean ± SD. The significance level was declared at ns, *P* > 0.05, *P < 0.05, **P < 0.01, and ***P < 0.001.

## Data Availability

All study data are included in the article.

## SUPPLEMENTAL MATERIAL

Fig. S1 jvi.00178-26-s0001.docx Screening of the rLLR-GSG-P2A-CPA optimal immunization strategy.

ASM does not own the copyrights to Supplemental Material that may be linked to, or accessed through, an article. The authors have granted ASM a non-exclusive, world-wide license to publish the Supplemental Material files. Please contact the corresponding author directly for reuse.
