# Supplementary material for: An oral rotavirus-vectored vaccine confers protection against Clostridium perfringens and rotavirus
Source: J Virol. 2026 Mar 26;100(4):e00178-26. doi: 10.1128/jvi.00178-26 (PMC13098247; doi:10.1128/jvi.00178-26)
Supplement: Fig. S1 — Screening of the rLLR-GSG-P2A-CPA optimal immunization strategy. [file jvi.00178-26-s0001.docx]

**
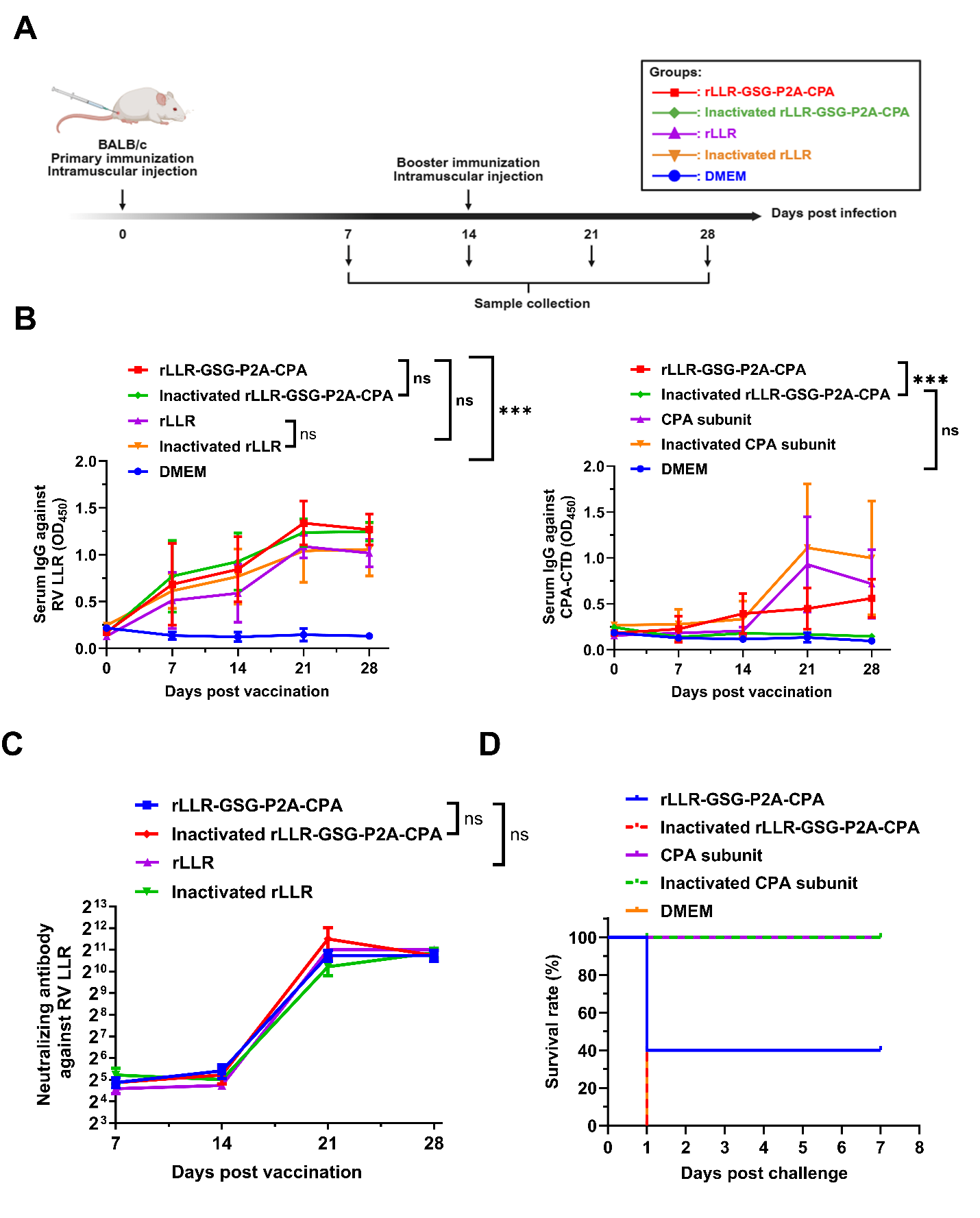
**

**Supplementary Material Fig. S1.** **Screening of the rLLR-GSG-P2A-CPA optimal immunization strategy.** (A) Immunization and sampling scheme. Five female BALB/c mice were immunized intramuscularly with infectious or inactivated rLLR-GSG-P2A-CPA (5×10^7^ TCID_50_) mixed with an oil adjuvant, ISA 15A VG, at a ratio of 85:15 (v/v). For the CPA subunit vaccine, mice were immunized intramuscularly with 20 µg of CPA protein mixed with an oil adjuvant, ISA 15A VG, at a ratio of 85:15 (v/v), and boosted with the same dose two weeks later. Sera were collected at the indicated timepoints. (B) Kinetics of serum IgG responses against rotavirus LLR and CPA-CTD. Antibody levels (OD₄₅₀) were measured by ELISA at 1:100 serum dilution. Data represent mean ± SD. Statistical analysis was performed by two-way ANOVA. Statistical significance is indicated as **p < 0.01 (C) Serum neutralization activities against rotavirus LLR. (D) Survival rates following lethal challenge with *C. perfringens* crude toxin at 21 dpv.
